# Supplementary material for: Whole-Transcriptome Analysis Highlights Adenylyl Cyclase Toxins-Derived Modulation of NF-κB and ERK1/2 Pathways in Macrophages
Source: Toxins (Basel). 2023 Feb 9;15(2):139. doi: 10.3390/toxins15020139 (PMC9967024; doi:10.3390/toxins15020139)
Supplement: Supplementary file 1 [file toxins-15-00139-s001.zip › toxins-2155727 SP ms.pdf]

Supplementary Material

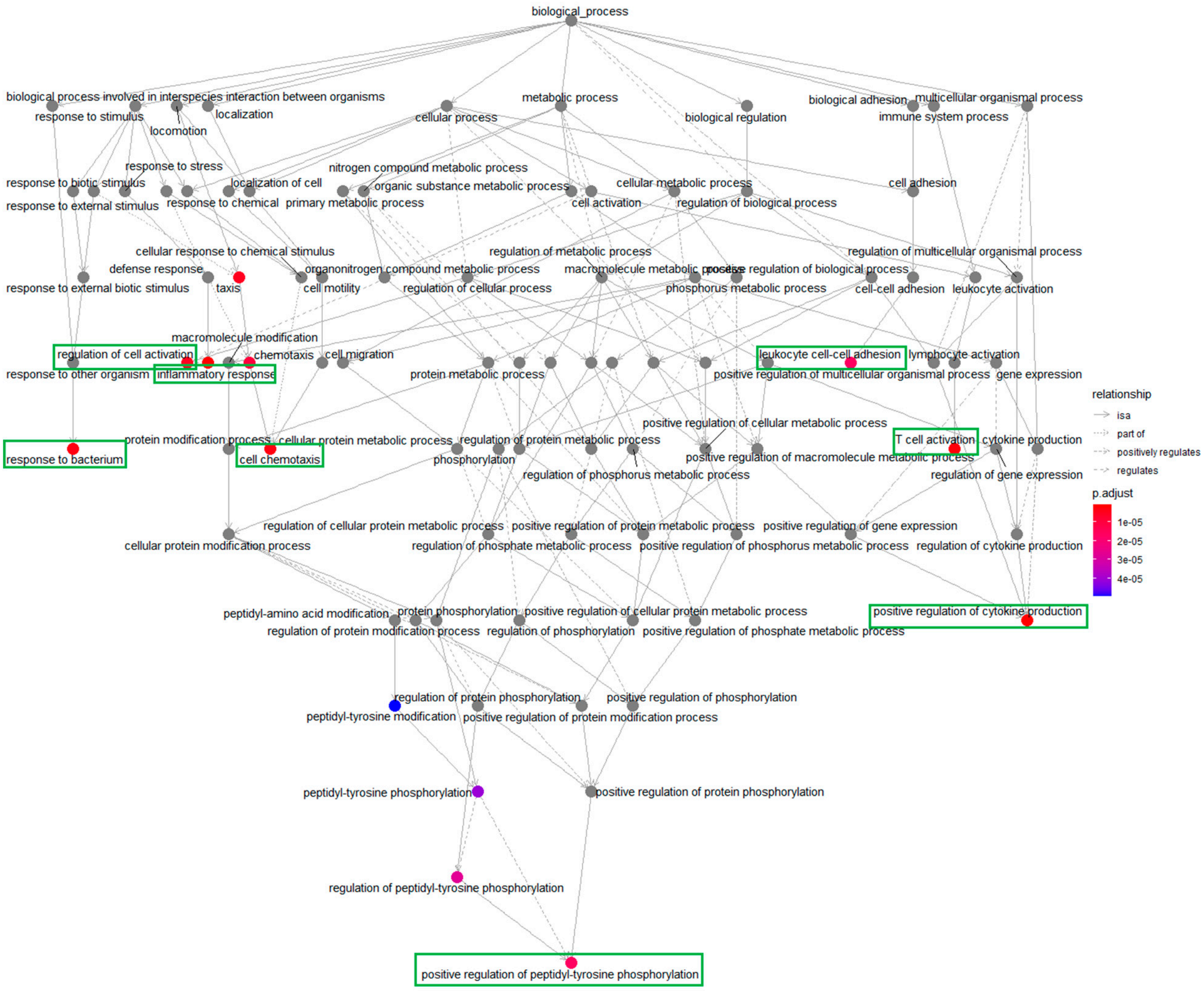

Figure S1. The relationship between the enriched biological process induced by exposure to ET(H351A) treatment in RAW264.7 macrophages. The most significant biological process Gene Ontology (GO) terms were structured in the form of directed acyclic graph to represent a network of complex correlations between 'child' and 'parent'. The more 'child' a GO term is, the more the term is related to a specific biological process. The most 'child' GO terms are indicated by green boxes.

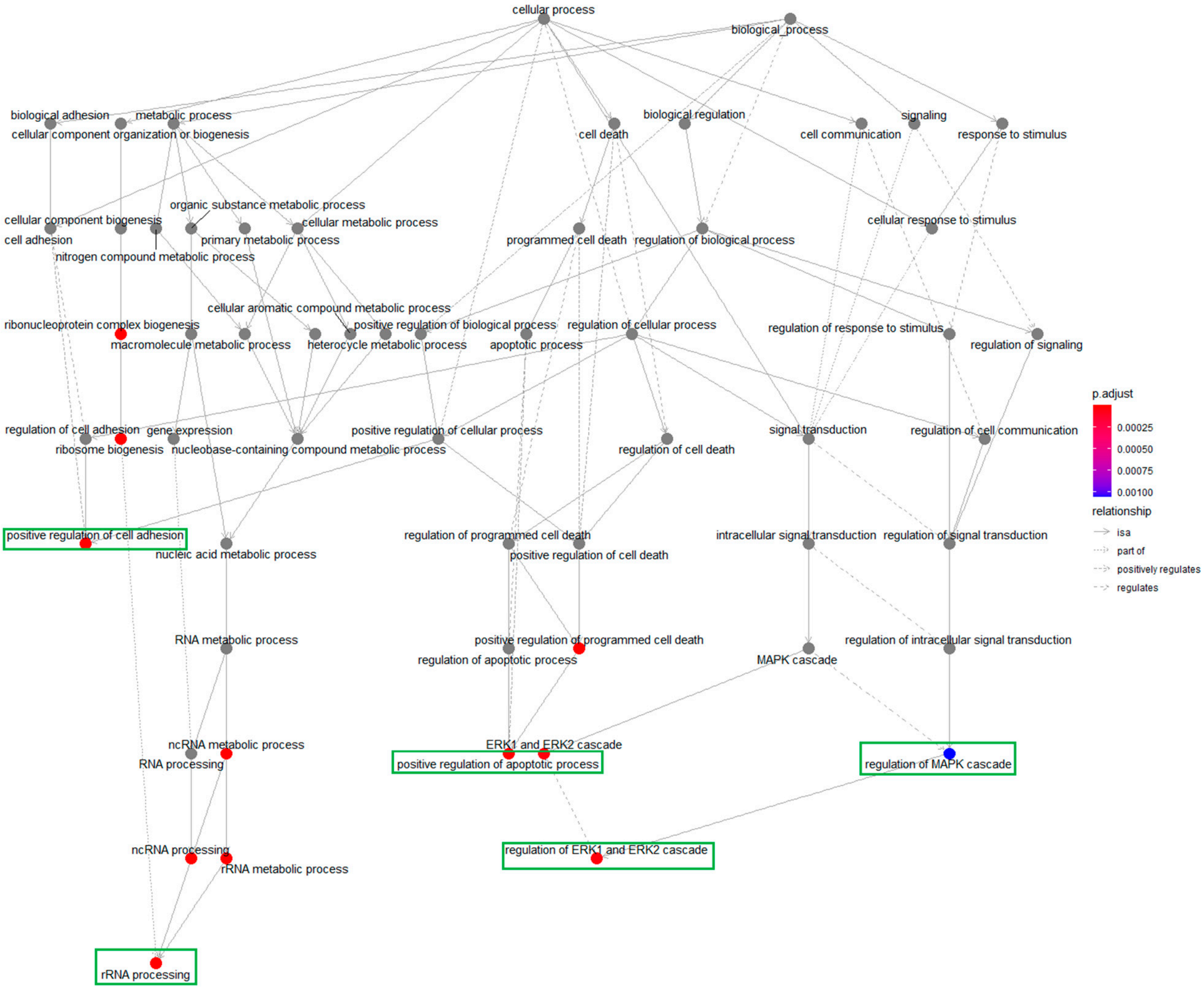

Figure S2. The relationship between the enriched biological process induced by ET treatment in RAW264.7 macrophages. The most significant biological processes among the Gene Ontology (GO) terms were structured in the form of directed acyclic graph to represent a network of complex correlation of 'child' and 'parent'. The more 'child' a GO term is, the more the term is related to a specific biological process. The most 'child' GO terms are indicated by green boxes.

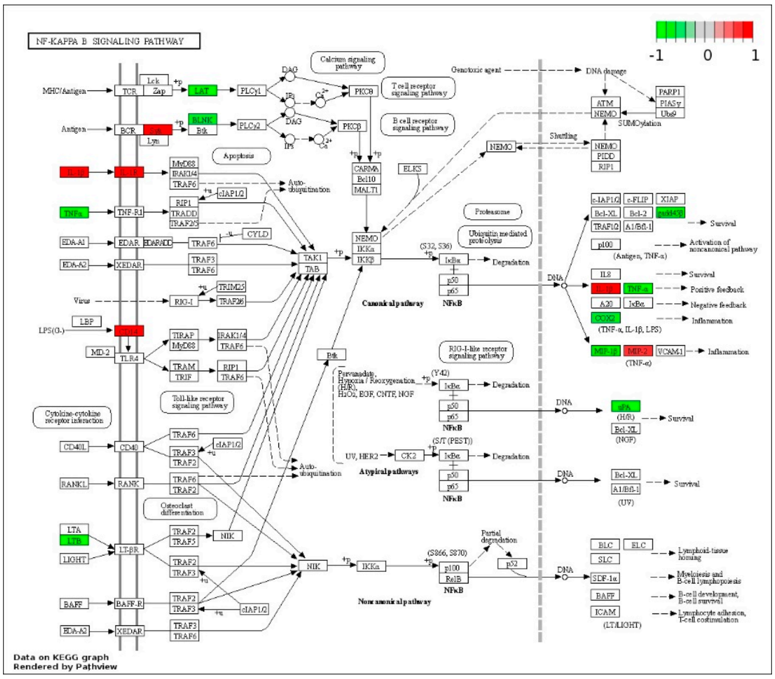

Figure S3. The differentially expressed genes induced by ET(H351A) treatment in RAW264.7 macrophages in the MAPK signaling pathway. The gene names highlighted by green indicate genes down-regulated by ET(H351A), and the gene names highlighted in red indicate genes up-regulated by ET(H351A).

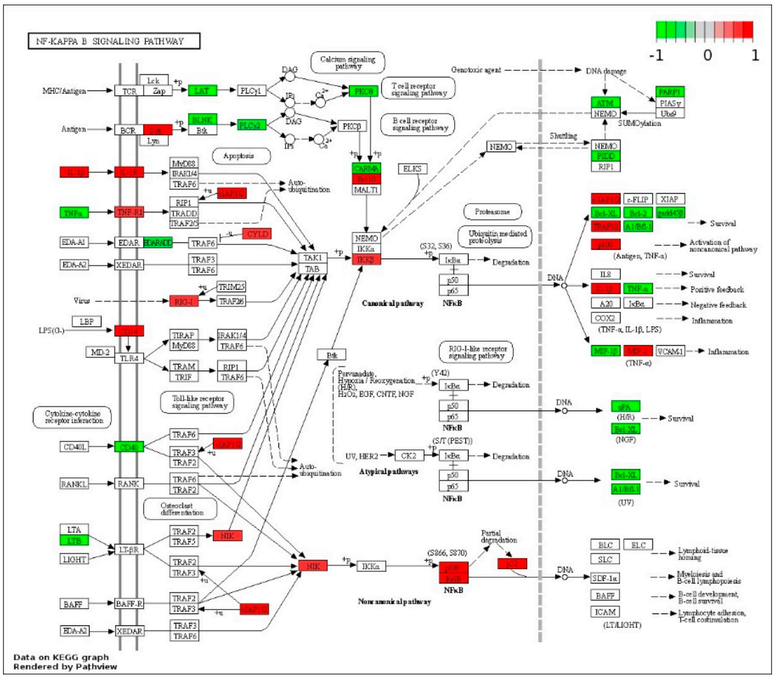

Figure S4. The differentially expressed genes induced by ET treatment in RAW264.7 macrophages affecting the MAPK signaling pathway. The gene names highlighted in green indicate genes down-regulated by ET, and the gene names highlighted in red indicate genes up-regulated by ET.

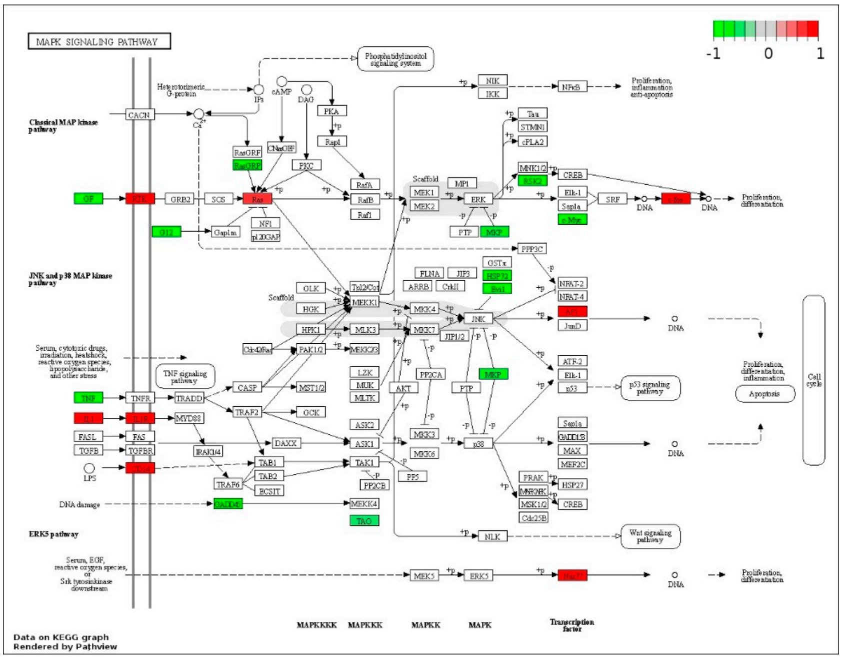

Figure S5. The differentially expressed genes induced by ET(H351A) treatment in RAW264.7 macrophages influencing the NF-κB signaling pathway. The gene names highlighted in green indicated that they were down-regulated by ET(H351A), while the gene names highlighted in red indicated genes up-regulated by ET(H351A).

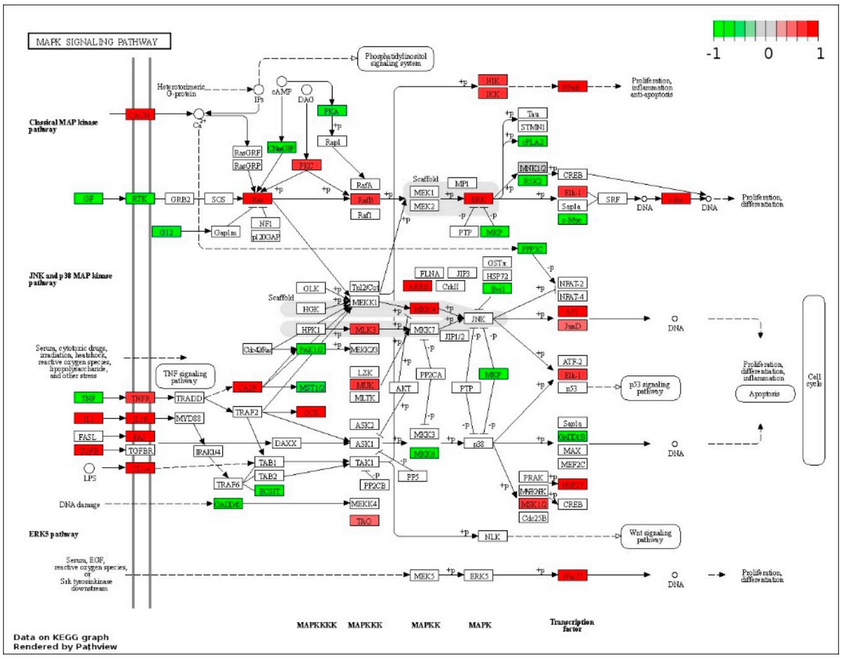

Figure S6. The differentially expressed genes induced by ET treatment in RAW264.7 macrophages involving the NF-κB signaling pathway. The gene names highlighted in green indicated genes that were down-regulated by ET, and gene names highlighted in red indicated genes that were up-regulated by ET.
